# Metabolic Potential of Halophilic Filamentous Fungi—Current Perspective

**DOI:** 10.3390/ijms23084189

**Published:** 2022-04-10

**Authors:** Weronika Śliżewska, Katarzyna Struszczyk-Świta, Olga Marchut-Mikołajczyk

**Affiliations:** Institute of Molecular and Industrial Biotechnology, Faculty of Biotechnology and Food Sciences, Lodz University of Technology, Stefanowskiego 2/22, 90-537 Lodz, Poland; weronika.slizewska@dokt.p.lodz.pl (W.Ś.); olga.marchut-mikolajczyk@p.lodz.pl (O.M.-M.)

**Keywords:** halophiles, filamentous fungi, biomolecules

## Abstract

Salty environments are widely known to be inhospitable to most microorganisms. For centuries salt has been used as a food preservative, while highly saline environments were considered uninhabited by organisms, and if habited, only by prokaryotic ones. Nowadays, we know that filamentous fungi are widespread in many saline habitats very often characterized also by other extremes, for example, very low or high temperature, lack of light, high pressure, or low water activity. However, fungi are still the least understood organisms among halophiles, even though they have been shown to counteract these unfavorable conditions by producing multiple secondary metabolites with interesting properties or unique biomolecules as one of their survival strategies. In this review, we focused on biomolecules obtained from halophilic filamentous fungi such as enzymes, pigments, biosurfactants, and osmoprotectants.

## 1. Introduction

Recently, the scientific interest in extremophilic microorganisms has increased largely because of their potential use in industrial biotechnological processes, where there is a need for enzymes capable of catalyzing reactions under various harsh conditions [1,2,3]. Those extremophiles could survive and thrive in stressful environments such as high or cold temperatures, acid or alkaline conditions, lack of nutrients, high salinity, and many others that were considered non-habitable. To overcome unfavorable conditions, microorganisms found special mechanisms, not found in non-extremophiles, allowing them to adapt. These could be highly flexible metabolisms, changes in the conformation of enzymes, production of secondary metabolites, and unique structural properties of their biomacromolecules [4,5,6]. While working with extremophiles and extremozymes, especially those obtained from saline environments, scientists mostly focus on bacteria, archaea, and even algae rather than on filamentous fungi that are still mostly undiscovered [7], even though extremophilic fungi show great potential for isolating new unusual compounds, especially since over 40% of active compounds obtained from microorganisms are produced by fungi [3,8].

Hypersaline environments are one of such extreme habitats where extremophilic microorganisms have been found. Those include not only seas, salt lakes, saline soil, salt deserts, salterns, and brines but also food products with a high salt content [9,10,11]. Therefore, halophiles are organisms that can survive in environments with high salinity, and occasionally, they even require salt to grow [12]. Halophilic microorganisms could be found in every domain, and they are mostly represented by bacteria, archaea, algae, and fungi [13]. All of them produce several interesting biomolecules with unique features that can be used in industrial applications.

Halophiles can have an industrial advantage as processes involving them in seawater can solve the problem of shortage of fresh water, avoid microbial contamination due to the presence of salt, and in the aftermath enable continuous processing without the need of sterilization [14,15]. For these reasons, industrial biotechnology using halophiles can compete with chemical processes as a technology allowing low consumption of fresh water and energy [15].

## 2. Classification of Halophilic Microorganisms

The most popular classification referring to all halophilic microorganisms is the one proposed by Kushner and Kamekura in 1988 [16]. It defines four categories based on the optimum growth of the microorganisms at different salt concentrations. The first category includes non-halophiles that require less than 0.2 M NaCl to grow, but if they can tolerate higher salt concentrations, they are considered halotolerant. The second category covers slightly halophiles, the ones growing best in salt concentrations between 0.2 and 0.5 M NaCl, which corresponds to the conditions prevailing in the seas (marine organisms). The next group is moderate halophiles that grow best in media containing 0.5 to 2.5 M NaCl, and finally extreme halophiles showing optimal growth above 2.5 M NaCl [16]. It is worth noting that halophiles are most often described as resistant to sodium chloride, but they can also refer to other inorganic salts and even soluble minerals that may be responsible, e.g., for the alkaline conditions making the fungus haloalkaliphilic [17,18,19].

## 3. Molecular Adaptations to High Saline Conditions

Osmotic and ionic stress related to high salinity prevents the survival of most microorganisms. Habitats with high salinity are also characterized by low water index and alkaline conditions and are a poor source of nutrients for the microorganisms living there [20]. To thrive in such harsh conditions, microorganisms create different adaptation mechanisms. One of the main ones is “salt in” strategy. It consists in accumulation by microorganisms of high concentrations of inorganic ions inside the cell to obtain osmotic balance [21]. Raising salt concentration in the cytoplasm is mostly a result of K^+^ ions because organisms from every domain exclude Na^+^ ions if possible due to their toxicity to several cell components, which include intracellular membranes and enzymes [22,23]. This strategy requires all the intracellular proteins to remain active and stable in presence of potassium chloride and other salts. To achieve it, the proteome of those organisms adapts to such conditions and is mainly made up of the acidic residues (aspartic and glutamic acid), which are located on the surface of the protein. They coordinate water molecules to form a protecting barrier around a protein that prevents dehydration and the precipitation of molecules from the solution [5,24]. Prokaryotes (bacteria and archaea) are the microorganisms that adapt to the “salt in” approach in environments where no fluctuating salinity conditions prevail [5]. Those organisms also are obligate halophiles with a demand to stay constantly in saline conditions [13].

Another strategy is known as “organic osmolytes” mechanism, “low-salt”, “salt-out” or “compatible solute” strategy [5,20,22]. Rather than accumulating inorganic salts, microorganisms use organic solutes to maintain the osmotic balance inside the cell. The compatible solutes include polyols, sugars, glycerol, ectoine, and dimethylsulfoniopropionate which do not interfere with the activity of enzymes [24]. Organic osmolytes could also be divided into three chemical categories: zwitterionic solutes (e.g., betaine, ectoine), noncharged solutes (e.g., sucrose, trehalose) and anionic solutes (e.g., β-glutamate, hydroxybutyrate) [21]. Metabolites obtained in “compatible solute” strategy are mostly extracellular, and therefore, they could be easily extracted [25]. This strategy needs the use of more energy than the “salt-in” strategy and is widespread among all halophilic microorganisms but is always related to halophilic Eukarya such as fungi [5]. 

The membrane structure of halophilic microorganisms plays an important role in adapting to saline conditions, protecting the cell from the harmful effects of changing salt concentrations and maintaining the osmotic homeostasis inside the cell by proper membrane fluidity [26]. Changes in the plasma membrane and the cell wall include structural modifications and the presence of pigments and/or hydrophobins [27,28,29]. High salinity increases the cell wall thickness and influences the change in lipid composition, which includes the number of sterols, the type of fatty acyl chains and the nature of the polar phospholipid head-groups [30]. Another factor influencing the properties of cytoplasmic membranes in halophilic microorganisms is the presence of pigments such as carotenoids and melanins which additionally screen out UV radiation and protect them from the damaging sunlight effects [21,31,32]. The black yeasts are a group of fungi that produce melanin in order to protect them from environmental stress. They are common in saline habitats and represent up to 80% of halophilic fungi [22]. The characteristics of those polymorphic fungi are meristematic, filamentous, or yeast-like growth and very thick cell wall with a distinct melanin layer [31,33]. Melanization of the cell wall helps to avoid water loss, leak intracellular compatible solutes, and maintain high fluidity of membrane, and therefore, fungi are able to survive in very high salinities [28]. The structure of the melanin layer depends on the level of salt concentration; in higher salinity, the porosity of the cell wall is increased which leads to leakage of glycerol and its higher extracellular level. In lower sodium chloride concentration, melanin granules form a thin continuous layer in the outer part of the cell wall [34]. Halophilic “black yeast” fungi are represented by *Hortaea werneckii* [28,34], *Trimmatostroma salinum* [35], *Aureobasidium pullulans* [36], and *Phaeotheca triangularis* [37].

## 4. Diversity of Halophilic Fungi

Mycobiota inhabiting natural saline environments consists of halotolerant and halophilic fungi, which are represented not only by previously known species but also by new and rare species [33]. For the most part, halophilic fungi, with the exception of a few obligate halophiles such as *Wallemia ichthyophaga*, do not require sodium chloride for growth, and they can adapt to a wide range of salt concentrations, from low concentrations characteristic of marine waters to nearly saturated NaCl solutions [38]. 

Research shows that halophilic fungi occur in a variety of hypersaline environments located almost all over the world, including Slovenia, Romania, Thailand, China, India, Brazil, and many others [9,39,40,41,42,43]. They were isolated from environments with different salinity levels such as marine environments (sea water, marine plants, and mangroves) [41,43,44], solar salterns that are installations for production of salt by evaporating sea water [39], natural salt lakes, salt mines [42], saline soil, salt deserts, sebkhas which are areas resulting from the evaporation of salt lakes characterized by a large variety of soluble salts [10], and salted foods and fermented products with a high salt content [45].

Their dominant representatives are yeast-like fungi and different related melanized species of the genus *Cladosporium*, anamorphic *Aspergillus* and *Penicillium*, the teleomorphic *Eurotium* and *Emericella*, filamentous species of genus *Wallemia*, *Alternaria,* and *Scopulariopsis* [46]. The majority of halophilic fungi have been classified into Ascomycota, and several species belong to Basidiomycota [44]. Within Ascomycota, the main genera include Capnodiales, Dothideales, and Eurotiales. Many halophilic fungi from those orders show a xerotolerant tendency, growing well in low a_w_ conditions beside halophilic capabilities [9]. Halophilic fungi which are important species of ascomycetes are *Aspergillus sydowii*, *Aspergillus versicolor*, *Aureobasidium pullulans*, *Hortaea werneckii*, *Penicillium chrysogenum*, *Phaetotheca triangularis*, and *Trimmatostroma salinum* [46,47]. Basidiomycota contains Trichonosporales, Sporidiales, and Wallemiales orders with significant species: *Wallemia ichthyophaga*, *Wallemia sebi*, and *Wallemia muriae* [48].

The best-known fungi considered to be model halotolerant and halophilic organisms include Hortaea werneckii, Wallemia ichthyophaga, Debaryomyces hansenii, Aureobasidum pullulans, and Trimmatostroma salinum [33].

## 5. Biomolecules Produced by Halophilic Fungi

Fungi from saline habitats are a source of an abundance of biocompounds with potential industrial applications, which have unique features such as salt tolerance, stability, and activity in presence of organic solvents and under low water activity conditions [29,49]. 

Halophilic fungi produce various enzymes, mainly known for their hydrolytic and ligninolytic properties [50,51]. Defense mechanisms against oxidative stress make halophiles a rich source of the compatible solutes or osmolytes including glycerol, arabinitol, erythritol, mycosporines, and mycosporine-like amino acids (MAAs) [27,34,52]. The saline environments represent the wide natural resources of fungal biosurfactants and surface-active proteins such as hydrophobins [53,54]. Halophilic microorganisms, including fungi, can also produce pigments. The black yeast-like and related melanized fungi accumulate melanin in their cells to protect the cell from harsh conditions [47]. Furthermore, they are considered producers of antimicrobial and anticancer compounds of pharmaceutical importance and antioxidants for food prevention and the cosmetics industry [20,43,55,56]. Halophilic fungi are still being discovered, and new compounds are described all the time.

### 5.1. Fungal Halophilic Enzymes

Enzymes obtained from halophilic filamentous fungi have the potential to find industrial applications under unfavorable conditions such as high salt concentration or presence of organic solvents which would normally inhibit enzymatic reactions [6,42]. They also maintain high stability and enzymatic activity at low water activity which can be as low as 0.75 [57]. Another feature of halophilic enzymes is their polythermophilicity, and it is common that in addition to salinity conditions, they can also resist various temperatures and a wide range of pH [15,58]. A characteristic of halophilic fungi is the fact that most of them produce extracellular enzymes, which makes their extraction under industrial conditions easier and more efficient compared to halophilic bacteria. Such enzymes are usually water-soluble and can adjust to lower water activity [59,60,61]. There are several specific adaptive mechanisms by which enzymes stand out.

Halophilic enzymes have more acidic amino acids and a smaller number of hydrophobic residues on the surface compared to hydrophobic residues [51,62,63]. That means they have a negative charge due to a lower percentage of small hydrophobic residues and basic amino acids (glycine, alanine, serine, threonine, lysine, and arginine), lower composition of amino acids with bulky hydrophobic side chains (phenylalanine, leucine, and isoleucine) compared to those mentioned above, and higher content of acidic amino acids (aspartic and glutamic acid) [6,51,64]. Mostly because of the negatively charged and very acidic amino acids on the surface of the enzymes, halophilic proteins can maintain functional conformation, preserve their solubility, reduce surface hydrophobicity, and prevent aggregation at high salt concentrations [24,65]. It also means that halophilic enzymes are more likely to form random-coil structures than α-helices [66].

Another way developed by halophilic proteins to maintain high solubility in high salt concentration was described as the “solvation–stabilization model”. The protein is surrounded by a solvation shell composed of a very high local concentration of solvent ions, which allows it to maintain balance by excluding small solutes by the solvation shell, thus avoiding protein precipitation [51,67]. Hydrated ions interact with acidic amino acids creating a negative surface which leads to the formation of shells of water protecting the protein from low water activity [49]. It allows proteins to remain stable, soluble, and active in very high salt concentrations [68]. In some cases, it also means the need for high salt concentrations, making halophilic proteins unstable in low-salt solutions [66].

The enzymes produced by halophilic fungi belong mainly to the classes of hydrolases (EC 3) and oxidoreductases (EC 1).

#### 5.1.1. Hydrolases

Halophilic hydrolases are one of the most commonly isolated enzymes from halophilic microorganisms, because of their potential use in biotechnological processes, requiring high stability and activity in the presence of organic solvents or at high salt concentrations [51,60]. Halophilic fungi are well known producers of amylases, lipases, cellulases, proteases, xylanases, pectinases, and others [42,69]. Those enzymes have industrial applications in various sectors such as biofuel production, bioremediation, food, cosmetics, detergent, and pharmaceutical processes [39,70,71,72]. Hydrolases obtained from fungi isolated from the saline environments are shown in Table 1.

#### 5.1.2. Oxidoreductases

Other enzymes from halophilic fungi which gained much attention are lignin-degrading enzymes (lignin peroxidases, manganese peroxidases, and laccases) with ability to degrade lignocellulose [58]. It was reported that marine fungi, mostly belonging to Ascomycota and Basidiomycota, carry out a ‘white-rot like’ role in marine environments [50,76]. It is possible that these ligninolytic enzymes play an important role in the decolorization of dyes, treatment of colored effluents, degradation of other organic pollutants, and bioremediation because of their ability to degrade saline and alkaline pollutants under both saline and non-saline conditions [50].

Examples of enzymes from the class of oxidoreductases produced by halophilic fungi are presented in Table 2.

#### 5.1.3. Other Classes of Enzymes

Marine fungi *Aspergillus oryzae* isolated from the brown alga *Dictyota dichotoma* has been reported to produce an extracellular alginate lyase (EC 4.2.2). This enzyme specifically cleaves at the β-1,4 glycosidic linkages between polymers consisting of 1,4-linked β-D-mannuronic acid (M) and α-L-guluronic acid (G) blocks of sodium alginate that produce homopolymeric blocks of polyM and polyG. Fungi were grown on medium containing 3% NaCl and 0,2% KCl, and the addition of NaCl (up to 150 mM) to assay medium increased the enzyme activity. The polyM and polyG blocks as obtained due to enzyme lyase from sodium alginate may have potential use for example in the biomedical industry [86].

### 5.2. Biosurfactants and Surface-Active Proteins

Biosurfactants are a diverse group of surface-active amphiphilic molecules produced by many microorganisms that have developed mechanisms enabling them to access hydrocarbons more easily. Microorganisms producing biosurfactants can be found everywhere, also among extremophiles that thrive in a wide range of temperatures, various pH, and salinity, which gives them industrial advantages over chemical surfactants in terms of lower toxicity and higher biodegradability [87]. Biosurfactants produced by halophilic microorganisms are capable of operating under growth limiting conditions. These compounds are characterized by the ability to work in the environment of physiological saline, hypersaline environments, and under increased temperature and pH. These characteristics make them valuable in industrial processes [88,89]. In the face of the antimicrobial resistance crisis, the antimicrobial and antiviral activity indicates the enormous potential of halophilic biosurfactants as antimicrobial agents in the field of biomedicine. These compounds can also be used in gene therapy and vaccine production [90,91].

Halophilic biosurfactants also show antioxidant activity and a strong anti-adhesive effect. The high stability of emulsions formed by halophilic biosurfactants also allows their use in the processes of mobilizing heavy crude oil, cleaning oil sludge from crude oil storage facilities. In addition, these compounds can be used to remove pollutants in highly saline wastewater or to increase the effectiveness of reclamation of saline environments polluted with hydrocarbons, including coastal bioremediation [92].

The group of molecules counted as microbial biosurfactants includes glycolipids, phospholipids, neutral lipids, lipopeptides, lipoproteins, fatty acids, polymeric, and particulate biosurfactants [54]. Some halophilic filamentous fungi have the ability to produce biosurfactants. Marine fungi *Aspergillus ustus* (MSF3) isolated from the marine sponge *Fasciospongia cavernosa* produced high yield of biosurfactant characterized as a glycolipoprotein [54]. *Aureobasidium pullulans* produced several different biosurfactants with 5-hydroxy-2-decenoic acid delta lactone (known as massoia lactone) as the main active compound [93]. 

Among surface-active proteins, hydrophobins have been described as the most powerful with the greatest ability to reduce the surface tension of water [94]. They are small (<20 kDa) cell-wall proteins produced by filamentous fungi, playing diverse roles in their growth and development [95]. Hydrophobins could only be found in fungi, not even yeasts contain genes encoding these proteins [27]. Their structure contains eight cysteine residues in a specific primary sequence pattern that form four disulfide bonds, stabilizing an amphipathic tertiary structure and driving hydrophobin self-assembly into amphipathic layers at hydrophobic–hydrophilic interfaces [96]. Those layers show good adhesion properties, ability to change the surface wettability, and protein adsorption behaviors [97]. Hydrophobins are divided into two classes. Class I hydrophobins form highly insoluble amyloid-like rodlets at interfaces, often undergoing a conformational change that can only be dissolved with strong acids. Hydrophobins of class II form a highly ordered two-dimensional crystalline monolayer at interfaces that can be easily dissolved with detergents, organic solvent solutions, or under high pressure [96]. Some of these features are exploited by fungi in hypersaline environments, where modulation of cell wall permeability can be of great importance in the presence of toxic salt ions constantly permeating the cell. With changes in the osmolarity of the environment, it is also useful to strengthen and stiffen the cell wall [95]. Comparison of halophilic fungi producing hydrophobins in saline environments with division into classes is summarized in Table 3. 

Another fungal surface-active biomolecules are cerato-platanins that are small cysteine-containing proteins (CP). They are mostly secreted into the culture filtrate but can also be found in the cell wall of fungal hyphae and spores. Solutions of CP lead to strong foam formation, and they self-organize on hydrophobic: hydrophilic interfaces into ordered, amphipathic layers. Proteins that belong to the cerato-platanins family have been found in some marine fungi such as *Aspergillus terreus* [87], *Trichoderma harzianum* [87], and *Trichoderma atroviride* [98].

### 5.3. Pigments

Carotenoids are well known pigments that are also produced as a response to stressful salinity conditions by halophilic microorganisms such as bacteria, archaea, algae, and yeasts, but in the case of halophilic fungi, they are not common and were only found in the species *Fusarium* sp. T-1 [99]. Strains were isolated from seawater collected from Japan and were grown with an artificial seawater as medium while the highest concentration of carotenoids was detected at 1/10 of an artificial seawater making this species rather halotolerant than halophilic. Found carotenoids were identified as β-carotene, γ-carotene, torulene, neurosporaxanthin, and neurosporaxanthin β-D-glucopyranoside [100].

Melanin, on the other hand, is the pigment that is widespread in halophilic fungi and has not been found in other halophiles. Melanins are high molecular mass pigments that typically are dark brown to black but could also have other colors. They are insoluble in aqueous and organic solvents because they are negatively charged and hydrophobic [101]. They are found in all organisms—microorganisms, plants, and animals. In fungi, melanins are present in cell walls as a distinct layer or extracellular as polymers formed by enzymes or autooxidation in the medium [37]. Most of fungal melanins from *Ascomycetes* and *Deuteromycetes* are termed DHN-melanins derived from 1.8-dihydroxynaphthalene (DHN) [37,102]. Those pigments help microorganisms to withstand extreme environmental factors such as a high UV radiation level, extreme temperatures, and osmotic stress [102].

As stated above, the halophilic filamentous fungi are mainly capable of synthesizing melanins; however, the species *Periconia* sp. has also been found to produce a rare blue pigment [103]. On the other hand, the quinone compounds (variecolorquinones), which showed a yellow color, were obtained from the halotolerant fungus strain *Aspergillus variecolor* [104].

### 5.4. Osmoprotectants

As mentioned earlier, halophiles produce organic osmolytes to counteract the damaging effects of high salt concentrations. Fungi mostly accumulate polyols (for example, glycerol, erythritol, arabinitol, xylitol, and mannitol), free amino acids and their derivatives, nitrogen-containing compounds such as glycine betaine, mycosporines, and mycosporine-like amino acids (MAAs) [34,46,105]. The compatible solutes produced by halophilic fungi are summarized in Table 4.

Glycerol is the major compatible solute, with great importance in maintaining positive turgor pressure at high salinity [34]. It is the simplest organic osmotic solute, has the smallest size, and its synthesis is the least complex, requiring less energy than the biosynthesis of any other compatible solutes detected in halophilic fungi [22]. Since the production of organic compatible solutes is energetically expensive, halophilic organisms attempt to reduce the energy generated for osmotic adaptation by producing smaller and simpler solutes [106]. In halophilic fungi, glycerol is produced from the glycolytic intermediate dihydroxyacetone phosphate by the NAD-dependent glycerol 3-phosphate dehydrogenase (Gpd) and glycerol 3-phosphatase (Gpp) as in *Debaryomyces hansenii* [107] or via the high-osmolarity glycerol (HOG) signaling pathway that is related to the adaptation of fungi cells to hyperosmotic stress detected in *Hortaea werneckii* and *Wallemia ichthyophaga* [28,108]. As reported, the amount of glycerol correlates with increase in salinity in the medium but also could be connected with the possible presence of other compatible solutes or ions [109]. 

Other compounds that could act as osmoprotectants are mycosporines and mycosporine-like amino acids (MAAs). They are small water-soluble molecules containing an aminocyclohexenimine or aminocyclohexenone unit substituted with amino acid residues [105]. MAAs play the role of sunscreen compounds protecting cells against damage dealt by harmful levels of UV radiation absorbing the wavelength range 310–365 nm, with maximum at 310–320 nm for fungi [52,110]. They also have other features, serving as antioxidant molecules scavenging toxic oxygen radicals or accumulating as compatible solutes in response to osmotic stress. In fungal species exposed to hypersaline conditions, MAAs act as supplementary osmotic solutes, supporting the cells to better tolerate the osmotic challenge [52].

### 5.5. Other Secondary Metabolites

Microorganisms growing in extreme environments produce unusual secondary metabolites for survival, growth, and communication purposes. It is known that fungi living in solar salterns, coastal saline habitats, deep-sea environments, and inhabiting the marine sponges and mangroves produce new bioactive molecules [113,114,115,116,117]. Some of them are of industrial and clinical importance demonstrating biological activity—antimicrobial, anticancer, and antioxidant. Examples of such biocompounds derived from halophilic fungi are shown in the Table 5. Halophilic fungi produce diverse bioactive compounds with antibacterial and antifungal activity against human [118] and plant [43] pathogens, but compounds with antiproliferative, antiangiogenic, anticancer, antibiotic, and antiviral activity also have a significant role [56]. Special attention is given to the current major global threats that are cancer and antimicrobial resistance to antibiotics, antivirals, and antimalarial drugs, and the still not fully explored halophilic fungi may be a source of unique secondary metabolites that will help to solve these problems [20]. An example of such promising compounds are spiromastixones, depsidone-based analogues found in a deep-sea fungi *Spiromastix* sp. which showed potent inhibitory effects against antibiotic-resistant forms of pathogenic bacteria [116]. One of the found compounds inhibited the growth of methicillin-resistant *Staphylococcus aureus* (MRSA) and *S. epidermidis* (MRSE) while another one inhibited vancomycin-resistant *Enterococcus faecalis* and *E. faecium* (VSE) making them a compound that could possibly be used as a treatment for multi-drug resistant bacterial infections. 

## 6. Industrial Applications of Halophilic Biocompounds 

Due to their unique features, biocompounds obtained from halophiles might have great industrial potential. Halophilic fungi, unlike halophilic prokaryotes, show the ability to grow both in salt-free conditions and in a wide range of salinity [33,46]. In turn, many representatives of halophilic archaea and bacteria, such as Halobacteriaceae, can grow at higher salt concentrations, but the lack of salt in the environment can lead to cell damage. Halophilic microorganisms can produce many different bioproducts, such as biopolymers, carotenoids, or enzymes, which are used in various industries, including food processing, as shown in the Table 6 [57]. However, there are significant differences between halophilic bacteria and halophilic fungi. Intracellular enzymes produced by haloarchaea, due to their osmotic equilibrium strategy associated with the accumulation of high concentrations of KCl inside the cell, may be more tolerant to salt and alkaline pH, but lose their activity in the absence of salt [13,124]. In turn, the enzymes of halophiles producing substances protecting against osmotic stress, i.e., fungi and some bacteria, can adapt to a wider range of extreme conditions, such as xerotolerance or thermostability and be a source of extracellular enzymes of better quality and quantity in biotechnological applications [25,78,125].

Production of numerous hydrolases and oxidoreductases resistant to salt presence and low water activity can be used in bioremediation processes and wastewater treatment. Fungi from saline environments are also a source of numerous ligninolytic enzymes, which could be useful in biomass conversion processes with the use of difficult to solubilize lignin materials [85]. 

The α-amylase from *Aspergillus gracilis* TISTR 3638 isolated from a solar saltern has shown higher activity in increasing salinity compared to commercial amylases, when used for waste water remediation, and it may find applications especially for industrial effluents contaminated with metallic ions [79]. Furthermore, other studies also investigated marine fungi for their ability to degrade polycyclic aromatic hydrocarbon (PAHs) in which *A. sclerotiorum* CBMAI 849 showed nearly 100% deletion of pyrene and over 76% of benzo[a]pyrene, making it attractive for bioremediation in saline conditions [126]. The halophilics *A. sydowii* EXF-12860 and *A. destruens* EXF-10411 have shown great results in removing 100% of xenobiotics—PAHs and pharmaceutical compounds (PhC) in wastewaters under salty conditions (>1M NaCl), so they could be used for biotechnological downstream processing of various industrial wastewaters [71]. This enzyme obtained from another halophilic fungi *A. penicillioides* TISTR3639 has potential application as an additive in the laundry detergent industry [78].

Halophilic fungi can conduct decolorization of dyes and purification of colored effluent. Ligninolytic enzymes produced by marine-derived fungi (*Tinctoporellus* sp. CBMAI 1061, *Marasmiellus* sp. CBMAI 1062, and *Peniophora* sp. CBMAI 1063) were able to decolorize up to 100% Remazol Brilliant Blue R dye (RBBR) under both saline and non-saline conditions [50].

Extensive research on biofuels, where several processes are carried out in the presence of high concentrations of NaCl, has focused the attention of researchers on halophiles [70]. Extracellular β-glucosidase from *A. sydowii* BTMFS 55 has been tested on different media for possible application in production of bioethanol, showing satisfactory results [74].

Biocompounds from halophilic fungi are promising for the pharmaceutical industry. Hydrophobins’ ability to form amphipathic membranes might find applications in the pharmaceutical industry for hydrophobic drug formulation and delivery. They can replace synthetic surfactants, which are used to improve drugs solubility in an aqueous environment and which have been shown to be immunogenic in immunocompromised patients as well as to increase drug stabilization [95,127]. Moreover, antimicrobial, anticancer, and antioxidant biological activities of secondary metabolites are expected to find applications as drugs in medicine [20].

Both melanin and MAAs are UV-absorbing compounds with antioxidant activities, and therefore, they could serve as sunscreen agents or potential anti-aging ingredients in cosmetics [52,128]. They have been found in many halophilic fungi, especially the black yeast, such as *Hortaea werneckii* [37,52].

## 7. Conclusions

Halophilic and halotolerant fungi, due to their adaptive mechanisms which they had to develop to survive in harsh salinity conditions, are the source of many compounds. Many of these biomolecules are unique compounds not found in other organisms or with features that give them advantages over compounds obtained from non-extreme environments. Not only enzymes, biosurfactants, compatible solutes, and pigments but also molecules with antimicrobial, antioxidant, and anticancer activities all could be found in fungi obtained from various saline habitats. The ability to remain stable and active at salt concentration in the presence of organic solvents and under conditions of low water activity makes biomolecules from halophiles applicable in industrial processes. Despite its many advantages and enormous potential, the mycobiota of the saline environment is still not fully explored, and therefore, it can still hide numerous biomolecules with unique properties. Therefore, further research on halophilic fungi should be carried out.

## Figures and Tables

**Table 1:** Hydrolases produced by halophilic filamentous fungi.

Organism	Enzyme	Salinity Growth Conditions with the Highest Enzyme Activity	References
*Aspergillus tubingensis* GR1	β-galactosidase	1.2 M NaCl	[40]
*Aspergillus flavus*	Cellulase	2.5 M NaCl	[39]
*Aspergillus gracilis*	Amylase, lipase, xylanase		
*Aspergillus penicillioides*	Amylase, xylanase		
*Aspergillus restrictus*	Cellulase, lipase, protease		
*Sterigmatomyces halophilus*	Lipase		
*Aureobasidium pullulans*	Protease	Seawater	[73]
*Scopulariopsis brevicaulis* LMK002	Endomannanase	1.7 M NaCl	[72]
*Scopulariopsis candida* LMK004			
*Scopulariopsis candida* LMK008			
*Verticillium dahliae* LMK006	Endomannanase, endoxylanase, cellulase		
*Aspergillus sydowii* BTMFS 55	β-glucosidase	0.17 M NaCl	[74]
*Halosarpheia fibrosa* CY685	Cellulase, xylanase	50% (*v*/*v*) artificial seawater	[75]
*Savoryella longispora* CY479			
*Halorosellinia oceanica* CY325	Endoglucanase, cellulose, xylanase		
*Hypoxylon* sp. CY326			
*Ascocratera manglicola* 9174	Cellulase, xylanase	1.5% (*w*/*v*) marine salts	[76]
*Astrosphaeriella striatispora* 7651			
*Cryptovalsa halosarceicola* 9142			
*Linocarpon bipolaris* 5790			
*Rhizophila marina* 9143			
*Penicillium* sp. K 1-7	Pectinase	0.5 M to 4.0 M NaCl	[77]
*Penicillium* sp. K 3-17			
*Penicillium* sp. K-5			
*Aspergillus* sp. Av 10	Xylanase, pectinase		
*Aspergillus* sp. Sh 86			
*Aspergillus niger* G 2-11			
*Aspergillus niger* S 87			
*Aspergillus niger* K6-11			
*Aspergillus penicillioides* TISTR3639	α-amylase	1.7 M NaCl	[78]
*Aspergillus gracilis* TISTR 3638	α-amylase	1.7 M NaCl	[79]
*Aspergillus sydowii* EXF-12860	Esterase	1.0 M NaCl	[71]
*Aspergillus destruens* EXF-10411		1.9 M NaCl	
*Chaetomium indicum*	β-1,3-Glucanase	0.2 M NaCl	[80]

**Table 2:** Oxidoreductases obtained from halophilic fungi.

Halophilic Fungi	Enzyme	Salinity Growth Conditions	References
*Marasmiellus* sp. CBMAI 1062	Laccase	Artificial seawater	[81]
*Peniophora* sp. CBMAI 1063			
*Flavodon flavus*	Lignin peroxidase, manganese peroxidase, laccase	Diluted seawater (1:1)	[82]
*Tinctoporellus* sp. CBMAI 1061	Lignin peroxidase	Artificial seawater	[50]
*Mucor racemosus* CBMAI 847	Lignin peroxidase, manganese peroxidases, laccase	0.5 M NaCl	[41]
*Aspergillus sclerotiorum* CBMAI 849	Lignin peroxidase, manganese peroxidases		
*Cladosporium cladosporioides* CBMAI 857			
*Aspergillus sydowii* EXF-12860	Peroxidase, laccase	1.0 M NaCl	[71]
*Aspergillus destruens* EXF-10411		1.9 M NaCl	
*Hortaea werneckii* EXF 225; MZKI B-736	Glycerol-3-phosphate dehydrogenase	1.0 and 3.0 M NaCl	[83]
*Wallemia ichthyophaga* EXF 994		1.8 and 4.5 M NaCl	
*Aspergillus sydowii* EXF-12860	Catalase, glutathione peroxidase and superoxide dismutase	1.0 M and 5.13 M NaCl	[84]
*Cadophora* sp. TS2	Peroxidase/phenol oxidases	0.5–2.0 M NaCl	[85]
*Emericellopsis* sp. TS11			
*Pseudogymnoascus* sp. TS12			

**Table 3:** Halophilic fungi as a source of hydrophobins.

Halophilic Fungi	Hydrophobins Class	Salinity Growth Conditions	References
*Aspergillus sydowii* BMH-0004	I	2.0 M NaCl	[27]
*Penicillium roseopurpureum* MUT 4892	I	0.5 M NaCl	[97]
*Acremonium sclerotigenum* MUT 4872	I	0.5 M NaCl	
*Roussoellaceae* sp. MUT 4859	I	2.0 mM NaCl	[53]
*Penicillium roseopurpureum* MUT 4892	I	2.0 mM NaCl	
*Acremonium sclerotigenum* MUT 4872	I	0.5 M NaCl	
*Penicillium chrysogenum* MUT 5039	II	2.0 mM NaCl	
*Myceliophthora verrucose* MUT 4878	II	0.25 M NaCl	
*Arthopyrenia salicis* MUT 4879	II	0.5 M NaCl	
*Wallemia ichthyophaga* EXF-994	Unclassified	1.7 and 5.1 M NaCl	[95]

**Table 4:** Organic osmolytes from halophilic fungi.

Halophilic Fungi	Compatible Solutes	Salinity Growth Range	Reference
*Hortaea werneckii* B-736	Glycerol, erythritol, arabitol, mannitol, MAAs	0–4.28 M NaCl	[34]
*Wallemia ichthyophaga* EXF-994	Glycerol, arabitol, mannitol	1.7–4.3 M NaCl	[111]
*Aspergillus sydowii* EXF-12860	Glycerol, erythritol, mannitol, arabitol, trehalose	1.0 M and 5.13 M NaCl	[84]
*Debaryomyces hansenii* Y7426	Glycerol, trehalose	0.6–3.0 M NaCl or KCl	[112]
*Debaryomyces hansenii* D 18335	Glycerol	0.7–2.0 M NaCl	[107]
*Phaeotheca triangularis* EXF-206	MAAs	1.7 M NaCl	[52]
*Trimmatostroma salinum* EXF-295			
*Hortaea werneckii* MZKI B-736			
*Aureobasidium pullans* EXF-150			
*Cladosporium cladosporioides* EXF-381			

**Table 5:** Different categories of bioactive compounds extracted from halophilic fungi.

Bioactivity	Halophilic Fungi	Compounds	Description	References
Antimicrobial	*Alternaria* sp.	Alterperylenol	Antibacterial activity against *Clavibacter michiganensis*	[43]
		Stemphyperylenol	Antifungal activity against *Alternaria brassicicola* and *Pestallozzia theae*	
	*Aspergillus flocculosus* PT05-1	New ergosterol derivative	Antimicrobial activity against *Candida albicans*, *Enterobacter aerogenes*, and *Pseudomonas aeruginosa*	[119]
		7-norergosterolide		
		3b-hydroxyergosta-8, 24(28)-dien-7-one		
	*Aspergillus protuberus* MUT 3638	Bisvertinolone	Antibacterial activity against *Staphyloccoccus aureus*	[118]
	*Phomopsis* sp. K38 and *Alternaria* sp. E33	Cyclo(D-Pro-L-Tyr-L-Pro-L-Tyr)	Antifungal activity against *Candida albicans*, *Fusarium graminearum*, *Gaeumannomyces graminis*, *Helminthosporium sativum* and *Rhzioctonia cerealis*	[113]
		Cyclo(Gly-L-Phe-L-Pro-L-Tyr)		
	*Spiromastix* sp. F19	Spiromastixones A−O	Antibacterial against gram-positive bacteria including *Bacillus subtilis*, *Bacillus thuringiensis*, and *Staphylococcus aureus*	[116]
		Spiromastixone J	Inhibitory effects against methicillin-resistant bacterial strains of *Staphylococcus aureus* (MRSA) and *S. epidermidis* (MRSE), and vancomycin-resistant bacteria *Enterococcus faecalis* and *E. faecium* (VRE)	
	*Trichoderma* sp. 05FI48	Trichoderins A, A1, and B	Antimycobacterial activity against *Mycobacterium bovis* BCG, *Mycobacterium smegmatis*, and *Mycobacterium tuberculosis* H37Rv	[114]
Antioxidant	*Aspergillus ochraceus* EN31	2-hydroxycircumdatin C	DPPH radical-scavenging activity	[120]
	*Keissleriella* sp. YS 4108	EPS2 (exopolysaccharide)	Site-specific and non-site-specific scavenging activity; scavenging activity against superoxide radical	[121]
	*Aspergillus wentii* EN-48	Methyl 4-(3,4-dihydroxybenzamido)butanoate	DPPH radical-scavenging activity	[122]
		5-O-methylsulochine		
		4-(3,4-dihydroxybenzamido)butanoic acid		
Anticancer	*Aspergillus* sp. nov. F1	Ergosterol	Potent cytotoxic activity to human tumor cell lines A549, Hela, BEL-7402, and RKO	[115]
		Rosellichalasin		
		Cytochalasin E		
	*Hypocrea vinosa* AY380904	SC2051 (phosphodiesterase inhibitor)	Inhibitory activity in the tyrosine kinase assay; inhibited HUVEC proliferation, migration, and tubule formation	[117]
		Hypochromins A		
		Hypochromins B		
	*Aspergillus flocculosus* PT05-1	New ergosterol derivative	Cytotoxicity against HL-60 and BEL-7402 cell lines	[119]
		7-norergosterolide		
		3b-hydroxyergosta-8, 24(28)-dien-7-one		
	*Plectosphaerella cucumerina*	Plectosphaeroic acid A, B and C	Inhibitory against indoleamine 2,3-dioxygenase (IDO)	[123]

**Table 6:** Application of halophilic fungal biocompounds.

Potential Applications	Biomolecules	Examples of Halophilic Fungi Producers	References
**Ecology**			
Bioremediation	lipase	*Aspergillus gracilis*, *Aspergillus restrictus*, *Sterigmatomyces halophilus*	[39,79,81]
Wastewater purification	amylase	*Aspergillus gracilis*, *Aspergillus penicillioides*	[39,81]
	lignin-degrading enzymes (lignin peroxidases, manganese peroxidases and laccases)	*Flavodon flavus*	[82]
Degradation of xenobiotics	lignin-degrading enzymes (lignin peroxidases, manganese peroxidases and laccases)	*Marasmiellus* sp., *Peniophora* sp., *Tinctoporellus* sp.	[81,129]
Dye decolorization	lignin-degrading enzymes (lignin peroxidases, manganese peroxidases and laccases)	*Flavodon flavus*, *Phanerochaete chrysosporium*, *Trametes versicolor*	[82,129]
**Renewable energy**			
Bioethanol production	cellulase	*Aspergillus flavus*, *Aspergillus restrictus*	[39]
	xylanase	*Aspergillus gracilis*, *Aspergillus penicillioides*	[39]
	β-glucosidase	*Aspergillus sydowii*, *Phaeotheca triangularis*	[60,74]
Biodiesel production	glycerol	*Hortaea werneckii*, *Wallemia ichthyophaga*, *Debaryomyces hansenii*, *Aspergillus sydowii*	[34,84,111,112]
**Food and feed industry**			
Processing of juices and wines	pectinase	*Penicillium* sp., *Aspergillus* sp., *Aspergillus niger*	[77,130]
Flavour enhancement	β- glucosidase	*Aspergillus sydowii*, *Phaeotheca triangularis*	[60,74]
Hydrolysis of lactose	β-galactosidase	*Aspergillus* spp., *Aspergillus tubingensis*	[40]
Fish sauce production	protease	*Aspergillus restrictus*, *Hortaea werneckii*	[39,60]
Feed additives	endomannanase	*Scopulariopsis brevicaulis*, *Scopulariopsis candida*, *Verticillium dahliae*	[72]
Foam stabilization	hydrophobin	*Roussoellaceae* sp., *Acremonium sclerotigenum*, *Myceliophthora verrucose*, *Arthopyrenia salicis*, *Penicillium roseopurpureum*	[53]
**Medicine, cosmetics, and pharmaceutical industries**			
Anti-aging ingredients	mycosporine-like amino acids	*Phaeotheca triangularis*, *Trimmatostroma salinum*, *Hortaea werneckii*, *Aureobasidium pullans*, *Cladosporium cladosporioides*	[52,128]
UV-absorbing agents	mycosporine-like amino acids	*Phaeotheca triangularis*, *Trimmatostroma salinum*, *Hortaea werneckii*, *Aureobasidium pullans*, *Cladosporium cladosporioides*	[52,105]
	melanin	*Hortaea werneckii*, *Phaeotheca triangularis*, *Trimmatostroma salinum*	[37,131]
Pharmaceutical additives	trehalose	*Aspergillus sydowii*, *Debaryomyces hansenii*	[84,112,132]
Drug carriers	hydrophobin	*Wallemia ichthyophaga*, *Acremonium sclerotigenum*, *Penicillium chrysogenum*, *Aspergillus sydowii*	[27,53,95,127]
**Consumer service (other industries)**			
Laundry detergents	α-amylase	*Trimatostroma salinum*, *Wallemia ichthyophaga*	[60]
	protease	*Aureobasidium pullulans*	[73]
Leather tanning	protease	*Aureobasidium pullulans*	[73]
Pulp bleaching in paper production	xylanase	*Hypoxylon* sp., *Halosarpheia fibrosa*, *Halorosellinia oceanica*, *Savoryella longispora*	[75,133]

## Data Availability

Not applicable.
